# Long-Term Application of Bioorganic Fertilizers Improved Soil Biochemical Properties and Microbial Communities of an Apple Orchard Soil

**DOI:** 10.3389/fmicb.2016.01893

**Published:** 2016-11-28

**Authors:** Lei Wang, Fang Yang, Yaoyao E, Jun Yuan, Waseem Raza, Qiwei Huang, Qirong Shen

**Affiliations:** Jiangsu Key Laboratory for Organic Waste Utilization, Jiangsu Collaborative Innovation Center for Solid Organic Waste Utilization, Nanjing Agricultural UniversityNanjing, China

**Keywords:** bioorganic fertilizers, soil microbes, apple yield, soil depth, composition

## Abstract

Soil biochemical properties and microbial communities are usually considered as important indicators of soil health because of their association with plant nutrition. In this study, we investigated the impact of long-term application of bioorganic fertilizer (BOF) on soil biochemical properties and microbial communities in the apple orchard soil of the Loess Plateau. The experiment included three treatments: (1) control without fertilization (CK); (2) chemical fertilizer application (CF); and (3) bioorganic fertilizer application (BOF). The high throughput sequencing was used to examine the bacterial and fungal communities in apple orchard soil. The results showed that the BOF treatment significantly increased the apple yield during the experimental time (2009–2015). The application of BOF significantly increased the activities of catalase and invertase compared to those in CK and CF treatments. The high throughput sequencing data showed that the application of BOF changed the microbial community composition of all soil depths considered (0–20 cm, 20–40 cm, and 40–60 cm), e.g., the relative abundance of bio-control bacteria (*Xanthomonadales, Lysobacter, Pseudomonas*, and *Bacillus*), *Proteobacteria, Bacteroidetes, Ohtaekwangia, Ilyonectria*, and *Lecanicillium* was increased while that of *Acidobacteria, Chloroflexi, Gp4, Gp6* and *Sphaerobacter* was decreased. The increase in apple yield after the application of BOF might be due to increase in organic matter, total nitrogen and catalase and invertase activities of soil and change in the bacterial community composition by enriching *Bacillus, Pseudomonas, Lysobacter*, and *Ohtaekwangia*. These results further enhance the understanding on how BOFs alter soil microbial community composition to stimulate soil productivity.

## Introduction

Maintaining soil sustainability is one of the most vital requirements for crop production in agricultural systems. Soil organic matter (SOM), soil enzyme activity and soil microorganisms are considered as important indicators of soil fertility and productivity because they determine soil biochemical properties (Bandick and Dick, 1999; Olk and Gregorich, 2006; van der Heijden et al., 2008). Furthermore, soil enzyme activity and microorganisms have been suggested as potential pointers of soil quality. Soil enzymes are involved in catalyzing various reactions and metabolic processes occurring in organic matter metabolism, maintaining soil structure, cycling nutrients, and producing energy for both microorganisms and plants (Kızılkaya et al., 2004; Khan et al., 2010). The effect of fertilization on soil enzyme activity and microbial composition has been intensively studied (Kandeler et al., 1999; Saha et al., 2008a; Zhao et al., 2014). Many studies showed that the application of organic fertilizers increases overall enzyme activity (Mäder et al., 2002; García-Ruiz et al., 2008; Moeskops et al., 2010), but activities of specific enzymes may change depending on the type of the organic fertilizer and the relative availability of nutrients. The enzymatic activity might be expected to enhance the availability of most limiting nutrients in order to meet microbial metabolic demands (Sinsabaugh et al., 2008; Allison et al., 2011). For instance, long-term N fertilization increased the activity of soil enzymes involved in labile C breakdown in conventionally managed agricultural soils (Bandick and Dick, 1999; Piotrowska and Wilczewski, 2012). Input of ordinary organic fertilizer can increase the soil enzymatic activity, but the response of enzymatic activity to the addition of bioorganic fertilizer (BOF) is not well understood.

Apple is one of the most important cash crops in China. As the first ranking country in the total growing area, China has taken the predominant position in the world apple production (Food and Agriculture Organization of the United Nations [FAO], 1999). Till 2012, the annual production of fresh apple was approximately 39.5 million tons and the cultivated area was 2.23 million ha, which occupied 52 and 46%, respectively, of the world apple production and planting area (Food and Agriculture Organization of the United Nations [FAO], 2014). In spite of the continuously increasing yield, the current average apple yields in China under farmer practices (17.96 tons ha^-1^ yr^-1^) remained low compared to that in other dominant apple producing countries, such as New Zealand, United States and some other European countries (e.g., France and Italy), where the annual yields ranged between 20 to 50 tons ha^-1^ yr^-1^ (United States International Trade Commission [USITC], 2010; Food and Agriculture Organization of the United Nations [FAO], 2014). The main constraints of apple yield in China are perceived to be poor soil properties (e.g., low soil nitrogen and organic matter contents) (Zhang et al., 2013a,b) that limit apple production. The SOM plays an important role in the functioning of agroecosystems (Loveland and Webb, 2003) and cropland fertility (Tiessen et al., 1994). Pan and Zhao (2005) and Pan et al. (2006) showed that soil organic carbon sequestration plays a significant role in increasing and stabilizing rice productivity. Understanding the controls of SOM on soil productivity and yield stability would provide a sound scientific basis for undertaking C inputs into soil under orchard ecosystem. Therefore, the use of BOF would be helpful to improve the apple production. The BOFs are not only a source of organic matter in soil which promotes plant growth by improving soil fertility, quality and nutrient use efficiency, but also provide nutrition to newly introduced microbes for the effective colonization and demonstration of plant growth promoting and bio-control traits. Previous studies showed that the useful effects of BOF on the soil physical, chemical and biological properties were directly related to the crop production (Rivera-Cruz et al., 2008; Shen et al., 2013).

Soil microorganisms play critical roles in regulating soil fertility and plant health and in the recycling of C, N, and other nutrients (He et al., 2005; Hartmann et al., 2009; Will et al., 2010). However, the associated general taxonomic responses to BOF addition are unclear. Soil microbial communities are often sensitive to BOF addition. Many reports found that the application of BOF significantly changed the soil microbial community composition (Shen et al., 2014; Wu et al., 2014; Zhao et al., 2014), with specific taxa, including *Proteobacteria* and *Acidobacteria*, often being very sensitive to the BOF addition (Shen et al., 2014). Similarly, Li et al. (2014) showed that the fertilization significantly changed the subsoil microbial community and composition. However, the influence of BOF on the soil microbial community composition and their implications for long term soil productivity and sustainability are not well understood or characterized.

The overall objective of this study was to examine that how the application of BOF affects the soil microbial (bacteria and fungi) community composition and enzyme activities in orchard ecosystem. This study is a part of larger project examining soil microbial composition and response of microbial taxa to BOF addition. There were two main hypotheses: First, BOF treatment would differ from CF and CK treatments in soil microbial community composition, enzyme activity and soil organic C, and these differences would depend on the quantity of input organic C; Second, the apple yield of BOF enriched soil would be influenced by the soil microbial taxa and soil enzyme activities.

The specific objectives of this study were: (1) to investigate the effect of BOF on soil organic carbon, soil enzyme activity and soil microbial community composition; (2) to explore soil microbial taxa responses to the application of BOF and (3) to evaluate the potential correlation among the underlying microbial taxa, soil enzyme activity and apple yield. We used Illumina-based sequencing approach to characterize the microbial community in the 0–60 cm soil depth under different fertilizer treatments in the Loess Plateau.

## Materials and Methods

### Site Description, Experimental Design and Soil Sampling

The field experiment station is located in the Linfen, Shanxi Province, China (35°08′18″N 111°13′15″E, 800 m a.s.l.). This region has a northern warm temperate zone, continental semi-arid monsoon climate, with an average annual temperature of 11°C and a mean annual precipitation of 494 mm. The soil type was classified as a typical calcareous soil in brown formed from Malan loess. The experiment was conducted in a 667 m× 667 m plots of appropriate-density (450 tree ha^-1^; 4 m distance between trees and 5 m between rows). The apple orchards were initially constructed in 1994 and the variety of apple was ‘Fuji.’ The experiment was established as a randomized complete block design with three treatments and nine replicates for each treatment in October 2008, and each plot consisted of 11 apple trees. The density of apple trees was 450 per hectare. Three treatments were as follows: (1) CK, control without fertilization; (2) CF, chemical fertilizer application and (3) BOF, bioorganic fertilizer application. In terms of CF treatment, two-third of the total chemical fertilizers (N 330 kg ha^-1^, P_2_O_5_ 378 kg ha^-1^ and K_2_O 354 kg ha^-1^) was applied as a basal fertilizer after harvest (October) and the remaining chemical fertilizers (N 165 kg ha^-1^, P_2_O_5_ 189 kg ha^-1^ and K_2_O 177 kg ha^-1^) were applied as a top dressing during the apple bud stage. In terms of BOF treatment, 11250 kg ha^-1^ of bioorganic fertilizer (N 281 kg ha^-1^, P_2_O_5_ 225 kg ha^-1^, K_2_O 169 kg ha^-1^, organic C 1631 kg ha^-1^ and functional microorganisms 5 × 10^7^g^-1^) and 1800 kg ha^-1^ of organic-inorganic mixed fertilizer (N 180 kg ha^-1^, P_2_O_5_ 126 kg ha^-1^, K_2_O 144 kg ha^-1^, organic C 209 kg ha^-1^) were applied as a basal fertilizer in October 2008 and 1440 kg ha^-1^ of chemical fertilizers (N 202 kg ha^-1^, P_2_O_5_ 230 kg ha^-1^ and K_2_O 216 kg ha^-1^) were applied as a top dressing during the apple bud stage. From 2009 to 2014, only 11250 kg ha^-1^ of bioorganic fertilizer was applied as a basal fertilizer in each October and the top dressing fertilization was the same as in 2008. The BOF was provided by the Jiangsu Provincial Key Lab for Organic Solid Waste Utilization, Nanjing Agricultural University, Nanjing, China. This BOF was enriched with *Bacillus subtilis* strain SQR9 after secondary fermentation and have cell counts of 5 × 10^7^ g^-1^ (Zhang et al., 2008). *Bacillus amyloliquefaciens* SQR9 has been reported excellent PGPR and bio-control strain (Cao et al., 2011). All other farm operations were according to the traditional farming methods. For the fertilizers application, the annular groove fertilization method was used at the depth of about 20–40 cm. The soil sampling in this study was performed in October 2015. The detailed soil sampling methods are presented in our previous study (Wang et al., 2016). Briefly, five apple trees were selected in each plot and soil samples at the depths of 0–20 cm, 20–40 cm, and 40–60 cm at a distance of 1.0 m from each tree at three different positions were collected. Then the samples for each depth were mixed together, and sieved (2 mm) to remove the above ground plant materials, roots and stones and divided into two subsamples: one was air-dried for soil biochemical analysis; the rest was stored at -80°C for DNA extraction and subsequent molecular analysis.

### DNA Extraction, PCR Amplification and Illumina-Based Sequencing

Genomic soil DNA was extracted from 0.5 g of frozen soil using the MoBio Power Soil^TM^ kit (Mo Bio, Carlsbad, CA, USA) according to the manufacturer’s instructions. The extracted DNA was evaluated using 1% agarose gel, and the concentration and quality (A260/A280) were determined using a Nano-Drop ND-2000 spectrophotometer (Nano-Drop, Wilmington, DE, USA). For the bacterial community analyses, the PCR primer pair (520f/802r) targets the V4 region of the 16S rRNA gene was used (Wang et al., 2016). For the fungal community analyses, we used PCR primers (ITS3/ITS4) to amplify the ITS2 spacer (Schoch et al., 2011). PCR reaction mixture was contained 13 μl PCR-grade water, 10 μl 5 Prime Hot Master Mix, 0.5 μl each of the forward and reverse primers (10 μM final concentration), and 1.0 μl genomic DNA (diluted 1:10 with PCR-grade water). Reactions were held at 94°C for 3 min to denature the DNA, with amplification proceeding for 35 cycles at 94°C for 45 s, 50°C for 60 s, and 72°C for 90 s; and a final extension at 72°C for 10 min for the 16S rRNA gene; and 4 min of initial denaturation at 94°C, with amplification proceeding for 30 cycles at 94°C for 30 s, 55°C for 30 s, and 72°C for 1 min; and a final extension at 72°C for 10 min for the ITS gene. After PCR amplification, the obtained products were purified using the QIAquick PCR Purification Kit (QIAGEN, Germany) and subjected to quantification using a Qubit^®^ 2.0 Fluorometer (Invitrogen, USA). Then, the purified amplicons were pooled in equimolar concentrations and employed for library construction using the NEB Next^®^ Ultra^TM^ DNA Library Prep Kit for Illumina (New England Biolabs, UK). The final quality and concentration of the libraries were checked using Agilent 2100 Bio-analyzer Instruments (Agilent Technologies Co. Ltd., USA) and determined using KAPA Library Quantification Kits (Kapa Biosystems, USA). All the library preparations for sequencing were performed using Illumina MiSeq platform (Wang et al., 2016).

Sequences were processed with the QIIME software package (Quantitative Insights Into Microbial Ecology) and UPARSE pipeline (Edgar, 2013). The reads were filtered by QIIME quality filter (Sun et al., 2014). The sequences retained for each sample, referred to as clean paired sequences, were analyzed following the UPARSE pipeline to pick up operational taxonomic units (OTUs) through making OTU table. Briefly, sequences with a quality score lower than 0.5 or length shorter than 200 nt and singletons were discarded, and the retained sequences were assigned to OTUs at 97% similarity, and chimeras were filtered. We picked a representative sequences for each OTU and used the RDP classifier (Caporaso et al., 2011) to assign the taxonomic data to each representative sequence.

### Soil Physicochemical and Enzyme Activities Analysis

Soil pH was determined with a glass electrode using a soil-to-water ratio of 1:2.5. SOM was determined with K_2_Cr_2_O_7_ oxidation-reduction titration method and Kjeldahl method was used for total nitrogen (TN) estimation. Soil nitrate were extracted with 0.01 M CaCl_2_ and determined with BRAN + LUEBBE AutoAnalyzer 3. Available potassium (AK) in soil was extracted with ammonium acetate and determined with flame photometry. Available phosphorus (AP) in soil was extracted with sodium bicarbonate and determined using the molybdenum blue method. Gravimetric water content (GWC) was determined by drying samples at 105°C for 48 h. The activities of the soil enzymes were assayed in four replicates with two non-substrate control using air-dried soil samples as described by Zhao et al. (2015). Urease and invertase activities were analyzed by the released ammonium and glucose equivalent, respectively. Catalase activity was measured by shaking for 20 min with H_2_O_2_ (3.5%) as a substrate, and the suspension was titrated with 0.1 mol L^-1^ KMnO_4_ solution. Catalase activity was expressed as 0.1 mol L^-1^ KMnO_4_ ml g^-1^ soil 20 min^-1^, whereas the other enzyme activities were expressed as products per gram of dry weight soil mass per incubation time (24 h).

### Statistical Analysis

Principal coordinates analysis (PCoA) was performed using the vegan package in R (Wang et al., 2016). The richness index, ACE and Chao1, diversity index for Shannon, soil physicochemical characteristics and the relative abundance of microbial taxa were analyzed by one-way analysis of variance using SPSS software version 20.0. Data were expressed as means with standard deviation (SD). Mean separation was conducted based on Duncan’s multiple range test. Differences at *P* < 0.05 were considered statistically significant.

### Accession number

Raw sequences for bacterial 16S rRNA genes and fungal IT2 space data have been deposited in the NCBI Sequence Read Archive (SRA) database with the accession number SRX2318970.

## Results

### Apple Yield

**FIGURE 1:**
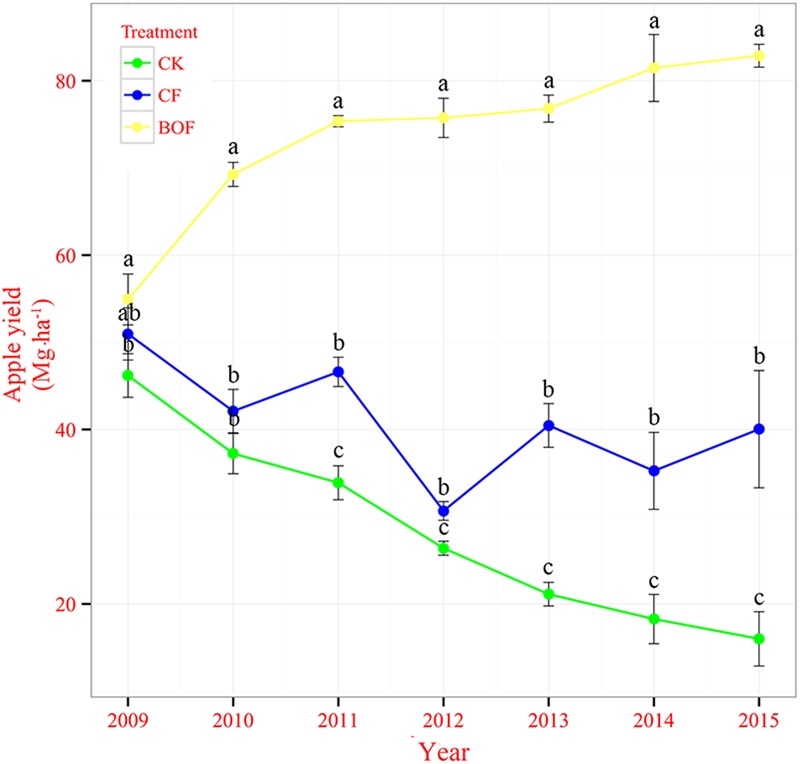
**Apple fruit yields from 2009 to 2013 under different fertilizer treatments.** CK, control without fertilization; CF, application of chemical fertilizer; BOF, application of bioorganic fertilizer and organic–inorganic mixed fertilizer. Values followed by different letters differ significantly (Duncan’s test, *P* < 0.05). Republished from Wang et al. (2016) with permission.

The application of fertilizers significantly increased the apple yield from 2013 to 2015 compared to CK treatment and apple yield from 2009 to 2013 was reported in our previous study (Wang et al., 2016). The apple yield was consistently increased from 2009 to 2015 in BOF treatment while there was a decreasing trend of apple yield in CK treatment. In the CF treatment, apple yield fluctuated during 2009–2015. In 2015, the apple yield in the treatment BOF was 82.88 Mg ha^-1^, which was 418 and 107% higher than that in the CK (16.00 Mg ha^-1^) and CF (40.04 Mg ha^-1^) treatments, respectively (**Figure 1**).

### Soil Physicochemical Characteristics

Fertilization had a significant effect on the soil physicochemical characteristics at all soil depths. Compared to the CK treatment, fertilization showed a significant (*P* < 0.05) decrease in soil pH, and the lowest pH was found in CF treatment at all soil depth. The highest NO_3_-N, AP and AK were found in CF treatment while the highest SOM and TN contents were found in BOF treatment at all soil depths tested (**Table 1**).

**Table 1:** Physicochemical analysis of soils from CK, CF and BOF treatments at different soil depths.

Soil depth (cm)	Fertilizer treatment	pH	SOM (mg g^-1^)	Total N (mg g^-1^)	NO_3_-N (mg kg^-1^)	AP (mg kg^-1^)	AK (mg kg^-1^)
	CK	8.43 ± 0.03 a	8.45 ± 0.11 c	1.36 ± 0.02 c	3.66 ± 0.61 c	6.96 ± 0.74 c	61.4 ± 1.2 c
0–20	CF	8.29 ± 0.02 c	12.26 ± 0.24 b	1.95 ± 0.04 b	16.39 ± 1.77 a	44.82 ± 1.67 a	114.9 ± 3.8 a
	BOF	8.36 ± 0.02 b	13.79 ± 0.62 a	2.19 ± 0.10 a	13.14 ± 1.10 b	30.48 ± 4.34 b	98.2 ± 2.8 b
	CK	8.46 ± 0.02 a	9.61 ± 0.27 c	1.57 ± 0.04 c	1.39 ± 0.27 c	1.43 ± 0.28 c	39.2 ± 2.3 c
20–40	CF	7.76 ± 0.02 c	13.40 ± 0.49 b	2.15 ± 0.08 b	54.20 ± 2.42 a	63.03 ± 2.41 a	134.0 ± 5.4 a
	BOF	8.07 ± 0.02 b	15.26 ± 0.50 a	2.44 ± 0.08 a	52.17 ± 2.08 b	24.16 ± 2.26 b	126.7 ± 3.0 b
	CK	8.56 ± 0.03 a	5.80 ± 0.25 c	0.94 ± 0.04 c	4.17 ± 1.47 c	2.17 ± 0.95 c	32.0 ± 1.7 c
40–60	CF	7.87 ± 0.02 c	7.58 ± 0.45 b	1.27 ± 0.12 b	48.95 ± 5.59 a	37.51 ± 1.60 a	163.7 ± 5.1 a
	BOF	8.01 ± 0.02 b	9.50 ± 0.13 a	1.51 ± 0.02 a	32.60 ± 5.43 b	11.11 ± 2.07 b	146.4 ± 1.7 b

*CK, control without fertilization; CF, chemical fertilizers; BOF, bio-organic fertilizers. Values followed by different letters differ significantly (Duncan’s test, *P* < 0.05).*

### Alpha-Diversity of Soil Microbial Community

For bacteria, at 0–20 cm soil depth, the BOF treatment showed a significant (*P* < 0.05) increase in the richness for Chao1 and decrease in the Good’s query coverage compared to CK and CF treatments. While at other soil depths, the differences of BOF treatment were non-significant with CF treatment. The richness for ACE was increased in the two fertilizer treatments (CF and BOF) compared to the CK treatment. The Shannon diversity was increased in BOF treatment compared to CK and CF treatments at 0–20 cm soil depth, but was decreased at 40–60 cm soil depth (**Table 2**).

**Table 2:** Microbial α-diversity indexes in different treatments at different soil depths.

		Bacteria				Fungi			
Soil depth (cm)	Fertilizer Treatment	Coverage (%)	Chao1	ACE	Shannon	Coverage (%)	Chao1	ACE	Shannon
	CK	94.73 ± 0.06 a	4659 ± 50 c	5408 ± 259 a	6.50 ± 0.09 b	99.24 ± 0.04 a	1230 ± 78 a	1277 ± 53 b	4.12 ± 0.61 a
0–20	CF	94.53 ± 0.06 b	4825 ± 64 b	5544 ± 173 a	6.59 ± 0.10 a	99.22 ± 0.03 a	1255 ± 57 a	1315 ± 87 a	4.24 ± 0.53 a
	BOF	94.40 ± 0.07 c	4982 ± 53 a	5451 ± 180 a	6.90 ± 0.05 a	99.20 ± 0.05 a	1260 ± 82 a	1403 ± 61 a	3.95 ± 0.50 a
	CK	94.88 ± 0.13 a	4533 ± 128 b	5239 ± 250 b	6.45 ± 0.07 b	99.29 ± 0.05 a	1224 ± 73 a	1228 ± 101 b	4.45 ± 0.45 a
20–40	CF	94.58 ± 0.11 b	4777 ± 92 a	5716 ± 129 a	6.53 ± 0.10 a	99.26 ± 0.06 a	1214 ± 117 a	1287 ± 107 ab	4.01 ± 1.00 a
	BOF	94.51 ± 0.10 b	4831 ± 75 a	5632 ± 313 a	6.52 ± 0.07 ab	99.24 ± 0.03 a	1194 ± 70 a	1326 ± 65 a	3.90 ± 0.64 a
	CK	95.06 ± 0.13 a	4401 ± 125 a	4930 ± 260 b	6.41 ± 0.12 a	99.35 ± 0.07 a	1103 ± 104 a	1154 ± 105 a	4.25 ± 0.43 a
40–60	CF	94.91 ± 0.12 b	4512 ± 100 a	5310 ± 168 a	6.35 ± 0.08 a	99.35 ± 0.05 a	1109 ± 84 a	1097 ± 77 a	4.21 ± 0.40 a
	BOF	94.87 ± 0.12 b	4530 ± 118 a	5425 ± 198 a	6.20 ± 0.21 b	99.31 ± 0.02 a	1061 ± 37 a	1178 ± 93 a	3.53 ± 0.15 b

*CK, control without fertilization; CF, chemical fertilizers; BOF, bio-organic fertilizers. Values followed by different letters differ significantly (Duncan’s test, *P* < 0.05).*

For fungi, the Shannon diversity and Good’s query coverage were decreased, while the richness for ACE was increased in BOF treatment compared to CK and CF treatments. The richness for Chao1 had no significant difference among the three treatments in case of fungi (**Table 2**).

### Microbial Community Structure

**FIGURE 2:**
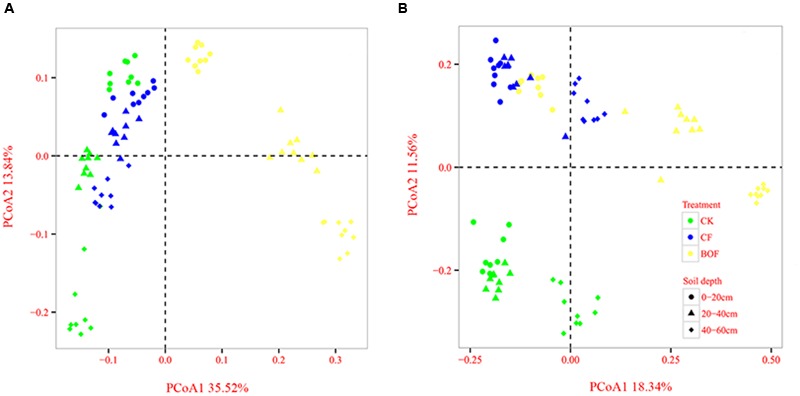
**Comparison of the bacterial (A)** and fungal **(B)** communities at 0–20 cm, 20–40 cm, and 40–60 cm soil depths under the different fertilizer treatments based on principal coordinates analysis of Bray-Curtis distances. CK, control without fertilization; CF, chemical fertilizers application; BOF, bio-organic fertilizers application.

Principal coordinates analysis of Bray–Curtis distances was performed to investigate the patterns of separation between microbial communities. The results clearly revealed that the soil microbial community varied among the different fertilizer treatments and soil depths. For the bacteria, the BOF treatment was separated distinctly from the CK and CF treatments along the first component (PCo1) at all soil depths, and the 0–20 cm soil depth was separated distinctly from 20–40 cm to 40–60 cm soil depths along the second component (PCo2) (**Figure 2A**). For the fungi, the CK treatment was clearly separated from the CF and BOF treatments along the second component (PCo2), and the 0–20 cm and 20–40 cm soil depths were separated distinctly from the 40–60 cm soil depth (**Figure 2B**).

### Microbial Community Composition and Ecological Significance of Selected Taxa

**FIGURE 3:**
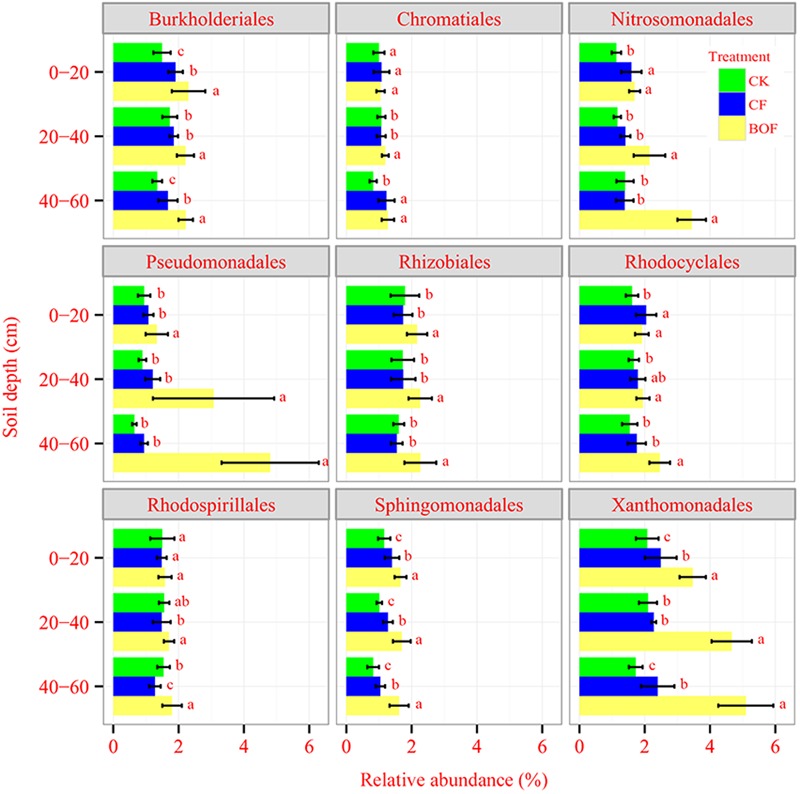
**Relative abundances of selected bacterial taxa (Order level) at different soil depths under different fertilizer treatments.** CK, control without fertilization; CF, chemical fertilizers application; BOF, bio-organic fertilizers application. Average relative abundance data from nine replicates were calculated as the ratio between the sequence type abundance and the total number of sequences. Values followed by different letters differ significantly (Duncan’s test, *P* < 0.05).

Fertilization had a significant impact on the microbial taxa distribution. The relative abundance of *Proteobacteria* and *Bacteroidetes* was increased; while that of *Acidobacteria, Chloroflexi* and *Planctomycetes* was decreased in BOF treatment compared to CK and CF treatments (**Supplementary Figure S1A**). The application of BOF had a significant effect on fungal taxa distribution, e.g., the relative abundance of *Ascomycota* was increased in BOF treatment at 20–60 cm soil depth and *Basidiomycete* was enriched in BOF treatment at both 0–20 cm and 40–60 cm soil depths. The relative abundance of *Chytridiomycota, Glomeromycota*, and *Zygomycota* was the highest in CK treatment compared to other two fertilizer treatments (**Supplementary Figure S1B**). Within phylum *Proteobacteria*, the relative abundance of Alpha-, Beta- and Gamma-*Proteobacteria* was significantly increased in BOF treatment compared to CF and CK treatments at all soil depths (**Supplementary Figure S2**). At the bacterial order level, the relative abundance of *Rhodospirillales, Rhizobiales, Sphingomonadales, Burkholderiales, Nitrosomonadales, Xanthomonadales*, and *Pseudomonadales* was higher in the BOF treatment compared to CK and CF treatments at 0–60 cm soil depth (**Figure 3**). At the genus level, for bacterial, the relative abundance of *Ohtaekwangia, Fluviicola, Bacillus, Nitrosospira, Nitrosococcus, Pseudomonas, Luteimonas, Lysobacter*, and *Steroidobacter* was increased, while that of *Gp4, Gp6, Nitriliruptor, Thermomicrobium, Sphaerobacter* and *Gemmata* was decreased in BOF treatment compared to CK and CF treatments (Supplementary Table S1). For fungi, compared to CK treatment, the relative abundance of *Chaetomium, Cryptococcus, Melanophyllum, Naumovozyma, Pannaria, Preussia*, and *Rhizopus* was depleted in the two fertilizer treatments. The relative abundance of *Hymenochaetales_unidentified_1, Mucor* and *Tetracladium* was higher in CF treatment compared to CK and BOF treatments and the relative abundance of *Ilyonectria, Lecanicillium* and *Minimedusa* was increased in BOF treatment compared to CK and CF treatments (Supplementary Table S1).

### Correlations between Microbial Community Structure and Environmental Variables

Mantel test was performed to compare Bray-Curtis distances of bacterial and fungal community structures and environmental parameters (**Table 3**). Our result showed that soil total N had a strong correlation with bacterial community structure (*R* = 0.2495, *P* < 0.001) while soil pH showed a strong correlation with the fungal community structure (*R* = 0.3785, *P* < 0.001).

**Table 3:** Mantel correlations between community structure and environmental variables.

	Bacteria	Fungi
pH	0.2020( < 0.001)	0.3785( < 0.001)
SOM	0.2486( < 0.001)	0.2980( < 0.001)
TN	0.2495( < 0.001)	0.2988( < 0.001)
NO_3_-N	0.1630( < 0.001)	0.3312( < 0.001)
AP	0.0538(0.09)	0.3306( < 0.001)

### Soil Enzyme Activity

The application of different fertilizers showed a significant effect on the soil enzyme activities at all soil depths. The activities of invertase and catalase were increased in the BOF treatment compared to CF and CK treatments. While, the urease activity was significantly higher in CF treatment compared to CK and BOF treatments at 20–60 cm soil depth (**Table 4**).

**Table 4:** Soil enzymatic activities in three treatments at different soil depth.

Soil depth (cm)	Fertilizer treatment	Invertase (mg g^-1^ h^-1^)	Urease (mg g^-1^ d^-1^)	Catalase (mg g^-1^ h^-1^)
	CK	12.56 ± 2.17 c	1.95 ± 0.07 b	9.61 ± 0.92 c
0–20	CF	10.55 ± 1.00 b	2.21 ± 0.06 a	11.70 ± 0.88 b
	BOF	16.20 ± 2.38 a	2.14 ± 0.08 a	17.00 ± 0.81 a
	CK	7.18 ± 1.26 c	1.86 ± 0.05 c	8.74 ± 0.97 c
20–40	CF	11.63 ± 1.58 b	2.21 ± 0.13 a	11.89 ± 1.29 b
	BOF	19.03 ± 1.93 a	2.02 ± 0.04 b	18.50 ± 1.35 a
	CK	1.67 ± 0.22 c	0.82 ± 0.06 c	7.78 ± 0.48 c
40–60	CF	7.83 ± 1.21 b	1.69 ± 0.11 a	10.95 ± 1.17 b
	BOF	11.02 ± 1.55 a	1.47 ± 0.06 b	16.42 ± 0.94 a

*CK, control without fertilization; CF, chemical fertilizers; BOF, bio-organic fertilizers. Values followed by different letters differ significantly (Duncan’s test, *P* < 0.05).*

### Relationship between Apple Yield and Selected Bacterial Taxa and Soil Enzyme Activity

**FIGURE 4:**
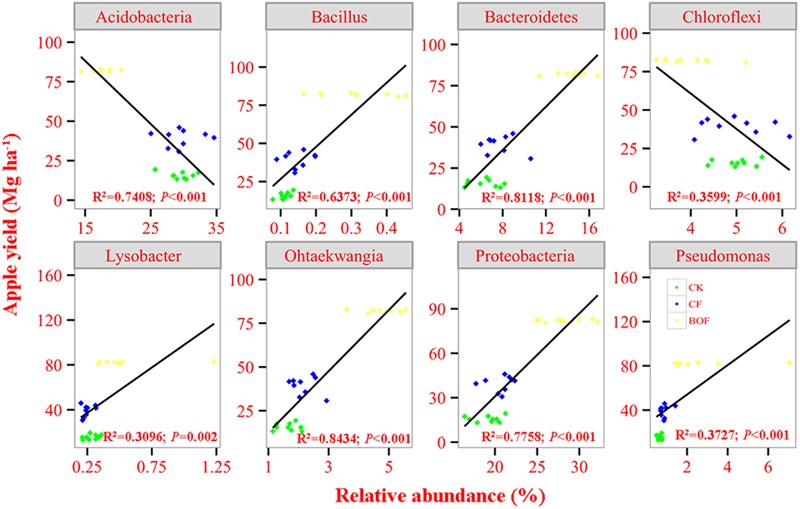
**Correlation analysis between the relative abundance of selected bacterial taxa and apple yield for treatments CK, CF and BOF.** CK, control without fertilization; CF, chemical fertilizers application; BOF, bio-organic fertilizers application.

Regression analysis showed that the relative abundance of *Proterobacteria* and *Bacteroidetes* was positively correlated while of *Acidobacteria* and *Chroloflexi* was negatively correlated with apple yield (**Figure 4**). Our result also found that apple yield had a positive correlation with four bacterial genera, i.e., *Bacillus, Ohtaekwangia, Lysobacter* and *Pseudomonas* (**Figure 4**). Moreover, the activity of invertase and catalase was also positively correlated with the apple yield (**Supplementary Figure S3**).

## Discussion

The present study attempts to investigate the effect of BOF on SOM, soil enzymatic activity and soil microbial (bacteria and fungi) community composition in an apple orchard ecosystem. The application of BOF increased the SOM, activities of catalase and invertase and changed the soil microbial (bacteria and fungi) community composition. The BOF treatment increased the apple yield compared to CK and CF treatments during 2009–2015 crop seasons. Many studies reported the beneficial effects of BOF on crop yields (Shen et al., 2013; Mosa et al., 2015; Xue et al., 2015). In this study, the apple yield was reached up to 81.47 Mg ha^-1^ and 82.88 Mg ha^-1^ in BOF treatment in 2014 and 2015, respectively, which was much higher than the average apple yield in the Loess Plateau and China. The apple yields of each year during the period of 2009–2015 were increased continuously in BOF treatment while fluctuated in CF treatment (**Figure 1**). The huge difference of apple yield between CF and BOF treatments could be primarily attributed to the improved soil biochemical properties.

The soil pH was changed greatly in both fertilizer treatments at all soil depths, which is consistent with the results of Li et al. (2014). The total nutrient content reflects the storage of soil nutrients, whereas available nutrient content reveal the dynamic balance between mineralization of the soil and the uptake by plants. The apple yield in CF treatment was lower than that in BOF treatment, which also explained that the available nutrients (AN, NO_3_-N and AP) were higher in CF treatment compared to BOF treatment. The continuous application of BOF led to an accumulation of SOM and TN, which ensured more stable and higher apple yields. SOM is important for plant nutrient transformation and is considered as a vital indicator of soil health and productivity. The SOM is also an important index of soil fertility because of its relationship to crop productivity (Vinther et al., 2004; Pan et al., 2009). In this study, the application of BOF significantly increased the SOM compared to CK and CF treatments. The increase in SOM level often leads to increased crop productivity. Thus, the BOF is a promising approach to develop more sustainable agriculture.

Soil enzymes are important in all biochemical processes that occur in the soil environment and are closely related to nutrient cycling, energy transfer, and environmental quality (Yao et al., 2006; Jiao et al., 2011); therefore, they have been used to predict the soil biological status and the effects of farm management practices on soil quality (Eivazi et al., 2003). The enzymes selected in the present study, i.e., invertase, urease and catalase, are related to soil C and N cycling (Dick, 1994). In the current study, the activities of invertase and catalase were higher in BOF treated soil than that in CK and CF treated soil, which was consistent with the results of Zhao et al. (2015). Our results showed that the application of BOF enhanced soil quality by improving the ability of soil to perform C cycling and transformation (as reflected by invertase activity). Catalase activity in soil is considered an indicator of aerobic microbial activity and has been related to both the number of aerobic microorganisms and soil fertility (García and Hernández, 1997). Therefore, the application of BOF enhanced soil fertility by improving the soil catalase activity. On the other hand, the chemical fertilizer treatment (CF) increased the urease activity which was coherent with the results of Liang et al. (2014). However, this finding was different from the results of Dick et al. (1988) and Bandick and Dick (1999), who reported that urease activity was decreased by the application of inorganic N. They hypothesized that the addition of the end product of the enzymatic reaction (NH_4_) could suppress enzyme synthesis (Saha et al., 2008b). In the present study, the positive effect of CF on urease activity might be because of the use of nitrate-based N fertilizer.

The fertilization has a significant influence on soil microbial community composition (Ge et al., 2008; Jangid et al., 2008). In the current study, the application of BOF increased the bacterial richness (Chao1) which was consistent with the results of Shen et al. (2015a). However, fungal Shannon diversity was lower in BOF treatment than that in CK and CF treatments. These results were in agreement with the previous reports that BOF application decreased the soil fungal diversity in the rhizosphere of cucumber (Zhang et al., 2013), cotton (Luo et al., 2010), watermelon (Zhao et al., 2011) and banana (Shen et al., 2015a). The principal coordinate (PCoA) (**Figure 2**) and permutational multivariate analysis (Supplementary Table S2) demonstrated that the fertilizer treatment was the more important factor than soil depth that influenced the soil microbial community composition. Zhao et al. (2014) also found that the fertilizer treatment was a more significant factor than sampling time to influence the soil bacterial community structure. The variance partitioning analysis (Supplementary Table S3) also showed that the fertilizer treatment explained higher unique variation in bacterial community structure relative to soil depth.

The results demonstrated that the BOF application increased the relative abundance of *Proteobacteria* and *Bacteroidetes*, and decreased that of *Acidobacteria* at all soil depths. These results were similar to the observation of a previous study (Shen et al., 2014). Among the *Proteobacteria, alpha-* and *gamma-Proteobacteria* were enriched in BOF treatment, which was in agreement with the observations of previous studies (Li et al., 2014). The finer taxonomic analyses revealed that the *Burkholderiales, Pseudomonadales* and *Xanthomonadales* showed increase in BOF treatment. These results were consistent with a previous work that identified disease-suppressive bacteria *alpha-* and *gamma-Proteobacteria* (*Pseudomonadaceae, Burkholderiaceae*, and *Xanthomonadales*) as the most dynamic taxa associated with the application of BOF (Mendes et al., 2011; Wu et al., 2014). Deeper taxonomic analyses revealed that the abundance of four genera, i.e., *Ohtaekwangia, Bacillus, Pseudomonas* and *Lysobacter* was increased with the BOF addition in soil. A previous study showed that the relative abundance of *Ohtaekwangia* was lower in BOF treatment than that in CK and CF treatments (Shen et al., 2014), this difference could be attributed to different soil types and fruit species response to BOF. Little physiological data exists for the genus *Ohtaekwangia*, because its ecological role in the soil is still unclear. Many previous studies demonstrated that *Bacillus* species play an important role in plant growth promotion (Raza et al., 2016) and disease suppression (Cao et al., 2011; Qiu et al., 2012). *Pseudomonas* species have also shown ability to promote plant growth and suppress a broad variety of pathogens such as *Phytophthora infestans* (Tran et al., 2007), *Agrobacterium tumefaciens* (Dandurishvili et al., 2011), and *Rhizoctonia solani* (Berta et al., 2005). Shen et al. (2015b) indicated that *Pseudomonas* genus was significantly enriched in suppressive soils. Many previous studies showed that the genus *Lysobacter* may identify members of this group effective in biological control based plant disease management (Christensen and Cook, 1978; Hayward et al., 2010). These results suggested that the application of BOF enriched the soil with beneficial microbes important for the positive plant-microbe interactions.

For fungi, *Ascomycota* and *Basidiomycota* were the most abundant fungal phyla at all soil depths, which was consistent with a previous study showing that *Ascomycota* and *Basidiomycota* were the most abundant phyla, accounting for more than 60% of the total sequences in a soil sample (McGuire et al., 2013; Schmidt et al., 2013). Many previous studies showed that environmental parameters shape the soil microbial community (Lauber et al., 2008; Rousk et al., 2010), especially soil pH, which has been confirmed in several studies to be the strongest factor shaping microbial community structure (Lauber et al., 2009; Rousk et al., 2010; Shen et al., 2012; Xiong et al., 2012). In the present study, mental test showed a robust correlation between Bray-Curtis distances and soil pH than other environmental parameters effect on the soil fungal community structure (**Table 3**). On the other hand, soil total N had the most influence on the soil bacterial community structure which was consistent with a previous study (Li et al., 2014).

## Conclusion

The activities of invertase and catalase were higher in the BOF treatment than CK and CF treatments at all soil depths. The application of BOF significantly changed the soil microbial community structure and composition, and enriched the important genera *Ohtaekwangia, Bacillus, Pseudomonas and Lysobacter* and decreased the abundance of *Gp4, Gp6, Sphaerobacter, Ilyonectria, Lecanicillium*, and *Minimedusa* at all soil depths. Soil total N and pH were found to be significantly correlated with the bacterial and fungal community composition, respectively. Further, the enrichment of *Proteobacteria, Bacteroidetes, Bacillus, Lysobacter, Ohtaekwangia*, and *Pseudomonas* was positively correlated with the apple yield, while that of *Acidobacteria* and *Chloroflexi* was negatively correlated with the apple yield. In general, the increase in apple yield after the application of BOF might be due to the fact that the application of BOF increased SOM, total N and activities of catalase and invertase and changed the bacterial community composition by enriching beneficial microbes that ultimately improved soil conditions to increase apple yield. This study further enhances the understanding on how BOF alters soil microbial communities to promote soil productivity.

## Ethic Statement

The authors declare that the research was conducted in conformity with the ethical standards of the field, the field studied did not involve human subjects or animals and biohazards.

## Author Contributions

LW performed the majority of the experiment. FY and YE performed part of the experiment. LW wrote the main manuscript text. QH and QS contributed insightful discussions. WR and JY reviewed and contributed to this manuscript.

## Conflict of Interest Statement

The authors declare that the research was conducted in the absence of any commercial or financial relationships that could be construed as a potential conflict of interest.

## Supplementary Material

FIGURE S1 **Relative abundances of selected bacterial (A)** and fungal **(B)** taxa (Phylum level) at different soil depths under different fertilizer treatments. CK, control without fertilization; CF, chemical fertilizers application; BOF, bio-organic fertilizers application. Average relative abundance data from nine replicates were calculated as the ratio between the sequence type abundance and the total number of sequences. Values followed by different letters differ significantly; ^∗^ indicate significant higher in BOF than CK and CF (Duncan’s test, *P* < 0.05).

FIGURE S2 **Relative abundances of four classes Alphaproteobacteria (A)**, Betaproteobacteria **(B)**, Deltaproteobacteria **(C)** and Gammaproteobacteria **(D)** at 0–20, 20–40, and 40–60 cm soil depths under different fertilizer treatments. CK, control without fertilization; CF, chemical fertilizers application; BOF, bio-organic fertilizers application. Average relative abundance data from nine replicates were calculated as the ratio between the sequence type abundance and the total number of bacterial sequences. Values followed by different letters differ significantly (Duncan’s test, *P* < 0.05).

FIGURE S3 **Correlation analysis between soil enzyme activity and apple yield for treatments CK, CF and BOF.** CK, control without fertilization; CF, chemical fertilizers application; BOF, bio-organic fertilizers application.
