# Supplementary material for: Long-Term Application of Bioorganic Fertilizers Improved Soil Biochemical Properties and Microbial Communities of an Apple Orchard Soil
Source: Front Microbiol. 2016 Nov 28;7:1893. doi: 10.3389/fmicb.2016.01893 (PMC5125012; doi:10.3389/fmicb.2016.01893)
Supplement: Supplementary file 4 [file Table_1.docx]

**Table S1** Relative abundances of selected bacterial and fungal taxa (genus level) at different soil depths under different fertilizer treatments. CK: control without fertilization; CF: chemical fertilizers application; BOF: bio-organic fertilizers application. Average relative abundance data from nine replicates were calculated as the ratio between the sequence type abundance and the total number of sequences. Values followed by different letters differ significantly (Duncan’s test, P<0.05).

| Soil depth (cm) |  | **0-20** |  |  | **20-40** |  |  | **40-60** |  |
| --- | --- | --- | --- | --- | --- | --- | --- | --- | --- |
| Treatment | **CK** | **CF** | **BOF** | **CK** | **CF** | **BOF** | **CK** | **CF** | **BOF** |
| **Bacterial (Genus)** |  |  |  |  |  |  |  |  |  |
| ***Gp4*** | 12.38±2.35 a | 14.19±3.45 a | 9.49±1.38 b | 13.34±1.46 a | 13.51±2.30 a | 6.87±0.99 b | 8.78±1.19 b | 13.29±1.51 a | 4.13±0.62 c |
| ***Gp6*** | 11.68±1.35 a | 11.07±3.51 a | 8.64±0.99 b | 11.11±1.23 a | 11.54±1.72 a | 7.07±0.96 b | 10.75±1.19 a | 12.04±1.87 a | 6.36±1.09 b |
| ***Steroidobacter*** | 1.31±0.27 b | 1.56±0.42 b | 1.96±0.29 a | 1.27±0.21 b | 1.38±0.10 b | 2.50±0.37 a | 1.01±0.15 c | 1.45±0.42 b | 2.65±0.55 a |
| ***Ohtaekwangia*** | 2.75±0.60 b | 2.89±1.09 b | 4.23±0.55 a | 1.66±0.36 c | 2.18±0.40 b | 4.82±0.63 a | 1.42±0.41 b | 2.01±0.46 b | 4.90±1.08 a |
| ***Nitrosospira*** | 1.13±0.14 b | 1.59±0.31 a | 1.69±0.17 a | 1.16±0.11 b | 1.41±0.15 b | 2.15±0.48 a | 1.40±0.26 b | 1.39±0.27 b | 3.44±0.44 a |
| ***Sphaerobacter*** | 1.08±0.15 a | 1.07±0.18 a | 0.84±0.06 b | 0.93±0.13 a | 0.98±0.25 a | 0.81±0.15 a | 0.80±0.07 b | 1.05±0.25 a | 0.94±0.12 ab |
| ***Fluviicola*** | 0.71±0.13 c | 1.10±0.53 b | 1.80±0.21 a | 0.39±0.10 c | 0.66±0.15 b | 1.45±0.23 a | 0.33±0.17 b | 0.48±0.11 b | 1.95±0.50 a |
| ***Thermomicrobium*** | 0.85±0.19 a | 0.79±0.23 a | 0.51±0.08 b | 0.72±0.07 b | 0.83±0.15 a | 0.44±0.08 c | 0.66±0.07 a | 0.75±0.13 a | 0.35±0.07 b |
| ***Nitriliruptor*** | 0.31±0.03 a | 0.31±0.03 a | 0.30±0.03 a | 0.60±0.10 a | 0.37±0.04 b | 0.33±0.06 b | 1.41±0.40 a | 0.54±0.07 b | 0.38±0.07 b |
| ***Gemmata*** | 0.87±0.21 a | 0.81±0.15 a | 0.61±0.06 b | 1.56±0.18 a | 0.96±0.15 b | 0.57±0.11 c | 2.33±0.33 a | 1.22±0.22 b | 0.51±0.11 c |
| ***Pseudomonas*** | 0.67±0.15 b | 0.80±0.11 ab | 0.94±0.34 a | 0.66±0.10 b | 0.88±0.23 b | 2.56±1.82 a | 0.46±0.04 b | 0.68±0.08 b | 4.33±1.44 a |
| ***Lysobacter*** | 0.22±0.04 b | 0.22±0.03 b | 0.37±0.04 a | 0.28±0.05 b | 0.25±0.04 b | 0.53±0.27 a | 0.23±0.04 b | 0.25±0.08 b | 0.84±0.09 a |
| ***Bacillus*** | 0.14±0.04 b | 0.12±0.02 b | 0.20±0.04 a | 0.11±0.02 b | 0.15±0.04 b | 0.31±0.10 a | 0.12±0.03 b | 0.12±0.03 b | 0.38±0.13 a |
| ***Nitrosococcus*** | 0.44±0.13 a | 0.34±0.06 b | 0.47±0.11 a | 0.34±0.06 b | 0.34±0.06 b | 0.53±0.10 a | 0.29±0.06 b | 0.24±0.03 b | 0.48±0.18 a |
| ***Luteimonas*** | 0.14±0.02 b | 0.17±0.03 b | 0.33±0.05 a | 0.17±0.03 b | 0.16±0.03 b | 0.56±0.11 a | 0.15±0.03 b | 0.20±0.04 b | 0.57±0.13 a |
| **Fungi (Genus)** |  |  |  |  |  |  |  |  |  |
| ***Ceratobasidium*** | 0.62±0.44 ab | 0.91±0.56 a | 0.44±0.10 b | 4.97±2.99 a | 1.26±1.25 b | 0.17±0.08 b | 1.80±1.10 a | 1.61±1.71 a | 0.16±0.04 b |
| ***Chaetomium*** | 1.69±0.78 a | 0.43±0.24 b | 0.26±0.06 b | 0.99±0.35 a | 0.59±0.42 b | 0.30±0.09 b | 1.79±1.22 a | 0.83±0.50 b | 0.48±0.09 b |
| ***Cryptococcus*** | 7.28±1.73 a | 4.12±1.02 b | 1.77±0.59 c | 5.18±2.01 a | 2.30±0.83 b | 0.58±0.32 c | 3.32±2.58 a | 1.84±0.74 ab | 0.40±0.07 b |
| ***Glomeraceae_unidentified*** | 2.25±3.28 a | 0.32±0.09 a | 0.39±0.23 a | 6.52±11.52 a | 0.29±0.18 a | 1.85±1.07 a | 4.47±12.37 a | 0.66±0.90 a | 0.22±0.20 a |
| ***Hymenochaetales_unidentified_1*** | 0.13±0.03 b | 4.29±3.75 a | 0.13±0.04 b | 0.20±0.13 b | 3.07±4.76 a | 0.03±0.02 b | 0.02±0.01 b | 0.96±1.40 a | 0.02±0.01 b |
| ***Ilyonectria*** | 0.78±0.24 b | 1.74±0.82 a | 2.19±1.02 a | 1.31±0.48 b | 1.86±0.95 b | 4.04±3.63 a | 2.32±1.04 b | 2.91±1.43 b | 7.87±2.11 a |
| ***Lecanicillium*** | 0.46±0.08 b | 0.47±0.12 b | 7.76±9.88 a | 0.42±0.08 b | 0.43±0.14 b | 6.90±4.93 a | 0.43±0.10 b | 0.40±0.09 b | 1.96±1.63 a |
| ***Melanophyllum*** | 6.79±14.56 a | 0.53±0.11 a | 0.67±0.21 a | 1.79±0.69 a | 0.49±0.18 b | 0.14±0.16 b | 1.64±1.49 a | 0.06±0.01 b | 0.09±0.02 b |
| ***Minimedusa*** | 1.47±1.00 b | 1.80±1.52 b | 8.01±5.32 a | 1.22±1.37 a | 1.22±1.40 a | 2.07±1.51 a | 0.91±1.76 a | 0.20±0.18 a | 0.22±0.15 a |
| ***Mucor*** | 0.07±0.02 b | 1.80±0.82 a | 0.09±0.02 b | 0.07±0.02 b | 1.75±2.04 a | 0.02±0.01 b | 0.03±0.07 b | 0.41±0.44 a | 0.01±0.00 b |
| ***Naumovozyma*** | 0.68±0.42 a | 0.34±0.29 a | 0.46±0.34 a | 1.46±1.29 a | 0.16±0.12 b | 0.64±0.19 b | 1.33±0.63 a | 0.77±0.37 b | 0.53±0.18 b |
| ***Pannaria*** | 0.36±0.19 a | 0.13±0.04 b | 0.07±0.02 b | 0.33±0.18 a | 0.10±0.05 b | 0.03±0.02 b | 0.25±0.22 a | 0.07±0.06 b | 0.01±0.00 a |
| ***Preussia*** | 5.16±3.87 a | 0.41±0.06 b | 0.41±0.11 b | 1.00±0.26 a | 0.40±0.11 b | 0.10±0.09 c | 1.73±1.50 a | 0.08±0.05 b | 0.11±0.05 b |
| ***Rhizopus*** | 1.46±0.99 a | 0.08±0.03 b | 0.09±0.03 b | 0.88±0.81 a | 0.10±0.08 b | 0.02±0.02 b | 0.40±0.32 a | 0.03±0.05 b | 0.01±0.01 b |
| ***Tetracladium*** | 2.62±1.88 b | 5.06±3.22 a | 3.96±1.54 ab | 3.12±1.33 a | 4.16±2.87 a | 2.24±1.12 a | 3.31±2.35 a | 3.91±5.24 a | 0.76±0.28 a |
