# Supplementary material for: Long-Term Application of Bioorganic Fertilizers Improved Soil Biochemical Properties and Microbial Communities of an Apple Orchard Soil
Source: Front Microbiol. 2016 Nov 28;7:1893. doi: 10.3389/fmicb.2016.01893 (PMC5125012; doi:10.3389/fmicb.2016.01893)
Supplement: Supplementary file 5 [file Table_2.docx]

**Table S2** Permutational multivariate analyses of variance of the Bray dissimilarity between bacterial and fungal communities.

| **Source** | **df** | **Abundence of bacterial OTUs** | |  | **Abundence of fungal OTUs** | |
| --- | --- | --- | --- | --- | --- | --- |
|  |  | **Sums of Sqs** | **Pseudo-F** |  | **Sums of Sqs** | **Pseudo-F** |
| **Fertilizer regime(FR)** | 2 | 1.97 | 29.14** |  | 4.74 | 18.47** |
| **Soil depth(SD)** | 2 | 0.8 | 11.89** |  | 2.75 | 10.72** |
| **FR×SD** | 4 | 0.8 | 5.95** |  | 2.22 | 4.32** |
| **Residuals** | 72 | 2.43 |  |  | 9.24 |  |
