# Supplementary material for: Long-Term Application of Bioorganic Fertilizers Improved Soil Biochemical Properties and Microbial Communities of an Apple Orchard Soil
Source: Front Microbiol. 2016 Nov 28;7:1893. doi: 10.3389/fmicb.2016.01893 (PMC5125012; doi:10.3389/fmicb.2016.01893)
Supplement: Supplementary file 6 [file Table_3.docx]

**Table S3** Relative influence of fertilizer treatment and soil depth on microbial community structure.

|  | **Respective contribution of contextual variables(% explained variance)** | |
| --- | --- | --- |
|  | **Fertilizer regime** | **Soil depth** |
| **Bacterial** | 27.89** | 10.94** |
| **Fungal** | 21.69** | 13.35** |
