# Supplementary material for: Long-Term Application of Bioorganic Fertilizers Improved Soil Biochemical Properties and Microbial Communities of an Apple Orchard Soil
Source: Front Microbiol. 2016 Nov 28;7:1893. doi: 10.3389/fmicb.2016.01893 (PMC5125012; doi:10.3389/fmicb.2016.01893)
Supplement: Supplementary file 7 [file Presentation_1.pdf]

Fig S1 Relative abundances of selected bacterial (A) and fungal (B) taxa (Phylum level) at different soil depths under different fertilizer treatments. CK: control without fertilization; CF: chemical fertilizers application; BOF: bio-organic fertilizers application. Average relative abundance data from nine replicates were calculated as the ratio between the sequence type abundance and the total number of sequences. Values followed by different letters differ significantly; * indicate significant higher in BOF than CK and CF (Duncan's test, $P<0.05$).

Fig S2 Relative abundances of four classes Alphaproteobacteria (A), Betaproteobacteria (B), Deltaproteobacteria (C) and Gammaproteobacteria (C) at 0-20, 20-40 and 40-60 cm soil depths under different fertilizer treatments. CK: control without fertilization; CF: chemical fertilizers application; BOF: bio-organic fertilizers application. Average relative abundance data from nine replicates were calculated as the ratio between the sequence type abundance and the total number of bacterial sequences. Values followed by different letters differ significantly (Duncan's test, $P<0.05$).

Fig S3 Correlation analysis between soil enzyme activity and apple yield for treatments CK, CF and BOF. CK: control without fertilization; CF: chemical fertilizers application; BOF: bio-organic fertilizers application.
